# Apoptosis and the Airway Epithelium

**DOI:** 10.1155/2011/948406

**Published:** 2011-12-13

**Authors:** Steven R. White

**Affiliations:** Section of Pulmonary and Critical Care Medicine, Department of Medicine, The University of Chicago, 5841 S. Maryland Avenue, MC 6076, Chicago, IL 60637, USA

## Abstract

The airway epithelium functions as a barrier and front line of host defense in the lung. Apoptosis or programmed cell death can be elicited in the epithelium as a response to viral infection, exposure to allergen or to environmental toxins, or to drugs. While apoptosis can be induced via activation of death receptors on the cell surface or by disruption of mitochondrial polarity, epithelial cells compared to inflammatory cells are more resistant to apoptotic stimuli. This paper focuses on the response of airway epithelium to apoptosis in the normal state, apoptosis as a potential regulator of the number and types of epithelial cells in the airway, and the contribution of epithelial cell apoptosis in important airways diseases.

## 1. Introduction

The airway epithelium is the first barrier and first line of host defense in the airway. Formerly considered a more inert barrier that “kept the outside out and the inside in,” it is now clear that epithelial cells participate in host defense and inflammation. The networks in which the epithelium participates indeed can orchestrate either or both, depending on whether these networks are activated normally or not.

A more classical view of epithelial responses to injury and inflammation emphasized the ability of the epithelium to respond to insults by secretion of water and mucous into the airways and mediator secretion (e.g., cytokines and chemokines) into the local environment and into the circulation. In this view, the epithelial layer responded to physical injury by a process that included, in order, phagocytic clearance of damaged cells and material, proliferation of new epithelial cells from surviving nearby stem cells, differentiation (phenotype shifting may be preferred) to new, required cell subtypes such as ciliated and mucous (goblet) cells, and restoration of barrier function [1]. Over the past two decades, it has become clear that proper protection and repair of the airway mucosa against sustained damage may also depend on the processes that control programmed cell death, that is, apoptosis.

Apoptosis is a tightly regulated process of nonnecrotic cell death that is critical for normal tissue and organ homeostasis. Cells undergo apoptosis through the activation of carefully regulated pathways that lead to their orderly shutdown and removal. In this paper, I examine the occurrence and function of apoptosis both in the normal airway epithelium and in the epithelium in several airways diseases. In the context of these diseases, epithelial cell apoptosis may be either a compensatory response, a pathogenetic consequence, or both. I limit this paper to discussion of central airways (tracheal and bronchial) epithelial cells with appropriate mention of small airway and alveolar epithelial cells where warranted and to the process of apoptosis, leaving aside other mechanisms of cell death such as necrosis and autophagy.

## 2. Studying the Airway Epithelium in Apoptosis

In examining apoptosis in the airway epithelium, it should be noted that several methods examine epithelium both *in situ* and in culture. Each of course has its limitations and strengths.

Collection of full-circumference airways is useful to examine epithelial morphology, damage, proliferation, and apoptosis in a setting that preserves the architecture of the full-thickness airway. Various morphologic and histologic strategies, including antibody labeling or electron microscopy, can be used. This method is limited to the study of specimens collected either by lung resection or at autopsy. Endobronchial biopsies can be obtained more easily (compared to open lung biopsy or autopsy) and allow for both one-time and, in some special cases, repeated sampling of airways, but crush artifact may limit the ability to interpret morphological changes in the mucosal layer [2]. Endobronchial brushings can collect epithelial cells for subsequent culture or harvest of RNA but provide no information about morphology.

The culture of airway epithelial cells likewise can be useful to elucidate mechanisms but has limitations. Primary cells typically have been grown in submersion culture, a method used for over four decades in many laboratories; these have a monomorphic appearance that resemble basal airway epithelial cells. Submersion culture can generate experiments quickly to test hypotheses and mechanisms but do not provide information about the morphology of epithelial subtypes and thus raises a concern about how applicable the results from these experiments are to the in situ state. Epithelial cells can be grown in an “air-liquid interface” (ALI), a more recent culture method that permits differentiation (a better term may be “phenotype shifting”) over a period of 2 to 5 weeks of culture into basal, columnar, and goblet cells that resembles a native epithelium [3, 4]. While more technically demanding, such cultures provide additional information about morphology and potential interactions between the epithelial subtypes. As with submersion culture, data from cells grown in ALI culture may not be fully applicable to the *in situ* airway. Finally, a number of airway epithelial cell lines have been generated over the past three decades. Several of these, such as the 16HBE14o- and 1HAEo- cell lines that are SV40 transformed from normal cells [5, 6] and the A549 lung adenocarcinoma cell line [7], have been popular in studies of epithelial cell apoptosis due to the ease of culture and the reproducibility of the cells over a number of passages. However, whether SV40 transformed or derived from cancer cells, it should be acknowledged that proliferation and apoptosis pathways and regulators may well be different in these cells versus primary cells and that cells in culture may not behave as epithelial cells *in situ* in their unique microenvironment.

One potential issue with the culture of epithelial cells is that (done properly) there are no other cell types in the culture system. While the response of pure epithelial cells to a stimulus is useful in reductionist-style experiments, examining interactions between different cell types may be just as important. To meet this concern, dual-culture systems grow epithelial cells on a filter either in submersion or in air-liquid interface suspended above a second cell type (e.g., fibroblasts) grown either on the underside of the filter or in the bottom of the culture container. Other dual-culture systems expose epithelial cells to bacteria (e.g., coculture or addition of *Pseudomonas aeruginosa*), with subsequent examination of apoptotic markers. Such biculture systems permit a limited examination of influences of these cells on epithelial cell apoptosis, or vice-versa.

Understanding the role of apoptosis in airway epithelium and interpreting the many studies done also require a clear awareness of the usefulness and limitations of each method of analysis. A recounting of apoptosis methods is well beyond this paper but two recent reviews outlining guidelines for the use and interpretation of assays to analyze cell death (apoptosis, autophagy, and necrosis) are useful reminders of the significant methodological and analytical issues [8, 9].

## 3. Apoptosis Mechanisms

Apoptosis is the orchestration of cell death by highly conserved genes in eukaryotes [10–13] that permits the removal of damaged or unneeded cells from tissue without releasing cellular contents that would otherwise damage surrounding cells or elicit an inflammatory response. It is tightly and carefully regulated in normal circumstances but may be inappropriately activated or suppressed in diverse disease states [12]. In contrast to cell necrosis, the principal features of apoptosis include shrinkage of the cell, condensation of the nucleus, DNA fragmentation, fragmentation of the cell cytoskeleton, and eventual formation of an apoptotic body that is either shed or phagocytized [14]. Several reviews examine general mechanisms of apoptosis and are recommended [9, 12, 15–17].

**Figure 1:**
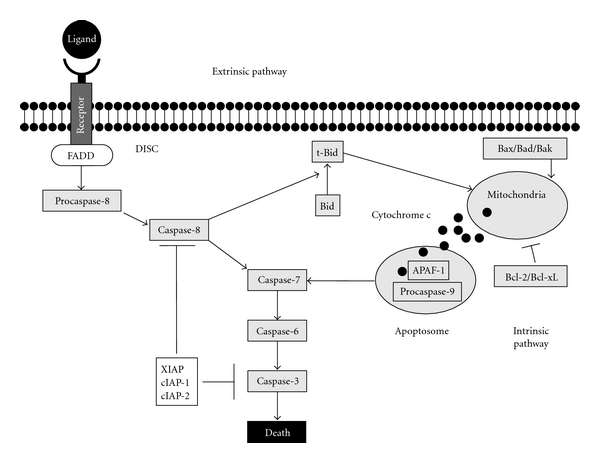
Extrinsic (receptor-mediated) and intrinsic (stress or oxidant-mediated) pathways that initiate apoptosis. Receptors such as CD95 and DR4 initiate apoptosis by interacting with death domain proteins such as FADD or TRADD, leading to the cleavage and activation of procaspase-8 with subsequent activation of downstream caspases and the initiation of the nuclear and cytoplasmic events that comprise apoptosis. In the intrinsic pathway, stress or oxidant-mediated injury leads to activation of proapoptotic BH3 proteins such as BAX or BAK; these then induce disruption of mitochondrial polarity leading to the release of cytochrome c, which binds APAF-1 in the “apoptosome,” leading to cleavage and activation of caspase-9. This then leads to downstream caspase activation. Cross-talk from the extrinsic pathway via the truncation of Bid (to t-Bid) can also elicit disruptions of mitochondrial polarity so that both pathways may be activated. Mitochondrial integrity is regulated by a series of related antiapoptotic (Bcl-2, Bcl-xL) and proapoptotic (Bad, Bax, Bak) proteins. The downstream caspase cascade can be inhibited by inhibitors of apoptosis such as XIAP, cIAP-1, and cIAP-2.

Multiple signals that initiate apoptosis including death receptors, cytokines, heat and oxidative stress, and ionizing radiation link to a signaling cascade of serine proteases known as caspases. These proteases are constitutively expressed as inactive zymogens that are cleaved when activated. Upstream caspases (e.g., caspase-8) work to collect receptor-mediated signals and initiate the death cascade, whereas downstream caspases (e.g., caspase-3) act upon targets in the both cell nucleus and cytosol (Figure 1).

Two distinct pathways exist for caspase activation. The first, type I, termed the “extrinsic” pathway because it is activated by extracellular signals, involves ligation of cell surface death receptors [11, 13, 18, 19], members of the tumor necrosis factor (TNF) superfamily, such as CD95 (also Fas or APO-1) [14, 20], TNFR1, DR3, DR4, DR5, DR6, EDAR, and p75NTR [21]. Each contains an exclusive, 80 amino-acid long domain, the “death domain,” essential to induce apoptosis [13, 21, 22]. When ligated, the receptors form homotrimers that recruit molecules into a “death domain.” Activation of these domains (e.g., interaction of Fas with Fas-associated death domain or FADD, or the interaction of TNFR1 with the TNR-receptor-associated death domain or TRADD) leads to formation of a death-inducing signaling complex (DISC) [23, 24] and subsequently caspase activation [25, 26].

The cognate death receptor ligands generally are comprised of a C-terminal extracellular portion which interacts with the death receptor, a transmembrane region, and an N-terminal domain. The best-described ligands are CD95L (FasL), that bind CD95, TNF-*α*, and the TNF-related apoptosis-inducing ligand (TRAIL).

CD95L is perhaps the best-characterized death receptor ligand. It was originally considered to be expressed mostly by hematopoietic cells such as lymphocytes and dendritic cells, but also is demonstrated in immune-privileged sites and in states of chronic inflammation, and not only mediates cell death but also serves as an effector molecule to establish immune privilege and enhance cell survival [21, 27]. Originally described as a transmembrane molecule, CD95L now is recognized to have a soluble variant cleaved from the cell surface by metalloproteases [28, 29]; alternately, it is packaged with other lysosomal proteins in lysosomes and transported to the plasma membrane where it is then released into the external environment [27]. Soluble CD95L binds its receptor with the same efficiency as the transmembrane form.

TNF-*α* has complex effects on airway epithelial cells and may both elicit and oppose apoptosis depending on context. It is a “proinflammatory” cytokine that induces several effects in airway epithelium, such as (of many examples) the expression of the ICAM-1 adhesion molecule [30–32] and IL-6 and IL-8 [33–35]. TNF-*α* binds two distinct receptors: TNFR1 and TNFR2 [36]. The former is responsible for the cytotoxic, including apoptotic, effects of TNF-*α*, whereas both receptors can interact with a series of adaptor proteins, the TNF-associated factors (TRAF). TRAF2 but not TRAF1 can initiate activation of the NF-*κ*B signaling cascade [37] to elicit IL-8 secretion [33, 34]. Disrupting NF-*κ*B signaling can enhance TNF-*α*-mediated apoptosis; likewise, activating NF-*κ*B signaling can suppress TNF-*α*-mediated apoptosis [38]. Thus, TNF-*α*-mediated cell death and inflammation exist in a balance that can be perturbed by several pathways.

TRAIL also is a member of the TNF superfamily [39]. TRAIL is a type II membrane protein that induces apoptosis in a variety of target cells. In some cell types, TRAIL binding to the death receptors DR4, DR5, and/or DcR2 may also activate the transcription factor NF*κ*B, leading to transcription of genes that actually antagonize death signaling pathways and promote inflammation [40, 41].

A second pathway that elicits apoptosis, type II or the “intrinsic pathway,” is activated not by receptors but by stressors such as oxidative stress, DNA damage, and starvation [14, 42] and has as its defining characteristic a requirement for increased mitochondrial permeability that then releases cytochrome c. Cytochrome c combines with APAF-1 to cleave and activate procaspase-9 in a complex termed the “apoptosome” [19, 43, 44]. The mitochondria thus may serve as a “central apoptotic executioner” to integrate multiple, diverse internal signals that indicate cellular damage. Activation of either pathway leads to cleavage and activation of common pathway caspases such as caspase-3 (Figure 1) and commits the cell to an apoptotic death [14].

Both pathways are regulated by a family of proteins known as the inhibitors of apoptosis (IAPs). Most of these suppress caspases either by binding the active catalytic site of effector caspases, by preventing dimerization of caspase-9, by sequestering mitochondrial proteins, or by stimulating degradation and ubiquitination of caspases [45]. The Bcl-2 family of prot1ns also heavily regulates the type II mitochondrial pathway. These proteins have a conserved Bcl2 homology domain that allows the family member to join to other members. The prosurvival members of the family (Bcl2, Bcl-XL, BclW, MCL1, A1, and BOO/DIVA) have multiple Bcl2 homology domains [14, 46]. In contrast, the proapoptotic protiens have a different number of Bcl2 homology domains: BAX and BAK, for example, each has three such domains, and both function to increase mitochondrial membrane permeability and thereby release cytochrome c [14, 47]. Other proapoptotic Bcl proteins (BIM, BID, PUMA) have an alternate Bcl2 homology 3 (BH3) domain that binds to and inhibits prosurvival Bcl2 family members, thus releasing BAX and BAK [48, 49]. A balance between prosurvival and proapoptotic Bcl2 proteins determines life or death, and each family member may be activated or suppressed differently depending on the cell stress or death activator.

## 4. Apoptosis Receptors and Receptor Ligands in Epithelial Cells

**Figure 2:**
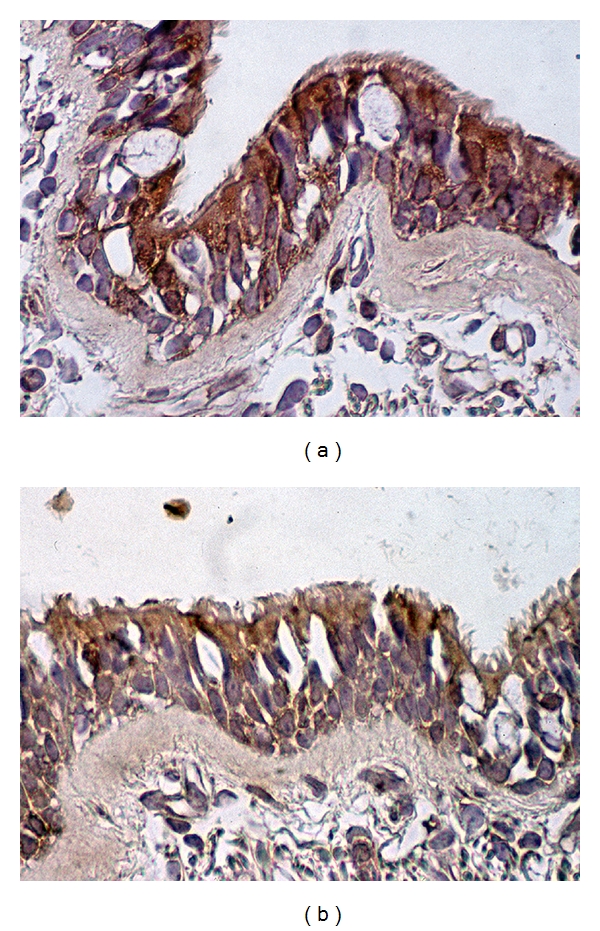
Expression of the CD95 receptor (a) and its ligand, CD95L (b), in a normal human airway using an immunoperoxidase method. Both basal and columnar cells stain for receptor and ligand (brown, with hematoxylin counter label). The appropriate controls (not shown here) for substitution of primary antibody do not label. Original magnification, 200x. From [20].

One death receptor clearly identified on airway epithelial cells is CD95. Our laboratory group first demonstrated this death receptor and the expression of its corresponding ligand CD95L (FasL), in primary central airway epithelial cells and cell lines in submersion culture and in airways collected from lung resection surgery [20] (Figure 2). Ligation of CD95 using the Ch11 antibody that cross-links the receptor activates caspase pathways and elicits cell death [20, 50, 51]. Both CD95 and CD95L subsequently have been demonstrated in airway epithelium in patients with cystic fibrosis [52]; CD95L expression is markedly increased in these airways, which was also demonstrated in an epithelial cell line, HTEC, with a CF genotype. These observations, including that the CD95 receptor is functional, have since been replicated; one such study suggests that epithelial cell apoptosis induced by CD95 ligation may be less important than that induced by TNF-*α* [53]. CD95 has been demonstrated in small airway epithelial cells (SAEC) in culture, and when compared to epithelial cells collected from larger, proximal airways, SAEC is more sensitive to CD95 ligation with a recombinant, soluble FasL [54]. CD95 expression also has been demonstrated in type II alveolar epithelial cells in humans and other mammals [55–58] and may be involved in fetal lung development [59].

 CD95L expression in human airway epithelium may serve, as it is postulated to serve in other immune privileged sites such as retina, brain, and testis, as an “immune barrier” [20, 60]. Airway epithelial FasL levels are increased in patients with severe asthma after steroid treatment, although it is possible that this increase in FasL levels reflects a more severe stage of disease [61]. One recent paper demonstrates that transmembrane FasL on epithelial cells is cleaved by matrix metalloproteinase (MMP)-7; this in turn is upregulated by the Th2-associated cytokine IL-13 [62]. As this MMP is increased in asthmatic airway epithelium, this study suggests a mechanism whereby increased CD95L in asthmatic airways might serve either to reestablish the immune barrier (by activating apoptosis in inflammatory cells) or to perpetuate mucosal damage (by activating apoptosis in epithelial cells).

These studies, taken together, make clear that both CD95 and its ligand are present and functional in airway epithelium both *in vivo* and in culture models and may be increased in expression in asthmatic epithelium.

As noted previously, TNF-*α* binds two distinct receptors: TNFR1 (also p60 TNFR) and TNFR2 (p80 TNFR). The former has an 80 amino-acid cystein-rich domain in its extracellular domain that can contain either a death domain, a TRAF binding domain, or a decoy domain [63]. The death domain interacts with the TNFR-associated death domain protein (TRADD), which in turn interacts with the Fas-associated death domain protein (FADD) to activate caspase-8 [37, 64]. In this way, TNFR1 shares the same downstream signaling pathway and machinery as CD95.

Remarkably few studies have been done to examine TNFR-pathway-driven apoptosis in airway epithelial cells and cell lines. Mitola et al. examined the effect of sputum sol phase collected from a cohort of patients with cystic fibrosis on apoptosis of primary human bronchial epithelial cells [65]. The TNF pathway was activated, but a direct link to TNF-*α* in the sputum sol phase was not demonstrated. Another recent study demonstrated that TNF-*α* stimulated caspase-3 activation and IL-8 expression in primary airway epithelial cells via phosphorylation of p38 mitogen-activated protein kinase (MAPK), an effected potentiated by concurrent exposure to nontypeable *Haemophilus influenzae* [66]. Activation of NF-*κ*B pathways and involvement of TRADD were not examined in this study. Other stress-activated protein kinases such as c-Jun N-terminal kinase (JNK) are well recognized to suppress TNF-*α*-stimulated apoptosis (reviewed in [67]), but this has not been studied in airway epithelium.

In addition to receptor-driven activation that directly initiates apoptosis, TNFR activation may modulate apoptosis by indirect mechanisms, in addition to any survival signals and inflammatory signals delivered by its activation of the NF-*κ*B signaling pathway. For example, treatment with TNF-*α* in primary proximal airway epithelial cells enhances subsequent apoptosis elicited by exposure to CD95L [54], demonstrating a clear potential interaction in these pathways. TNF-*α* treatment in the H441 and A549 lung adenocarcinoma lines elicits gene expression of both *TRAF1* and *cIAP2* and that both were increased in the lungs of infants with fatal bronchopulmonary dysplasia [68]. These signaling interactions are complex but suggest potential therapeutic strategies in manipulating death versus survival pathways in TNFR-mediated signaling.

The expression of TRAIL, a ligand for several death receptors, is increased, as demonstrated by immunohistochemical labeling, in the epithelium of endobronchial biopsies collected from asthmatic airways [69]. TRAIL expression is also noted in Th2 cells [70]. However, in contrast to the clear presence of CD95, no published studies have demonstrated the presence of potential partner death receptors, including DcR1, DcR2, DR4, or DR5, in airway epithelium in normal human airways, though one paper demonstrates both DR4 and DR5 in guinea pig airways [71], and one paper demonstrated the R1 and R2 receptors for TRAIL in nonbronchoscopically obtained epithelial cells from children suffering from RSV infection accompanied by respiratory failure and mechanical ventilation [72]. TRAIL may bind one of the TNFRs that are induced during diseases processes, which then may activate cell death pathways via relatively novel signals that include phosphorylation of p38-MAPK [66]. The relative expression of and signaling pathways connected to these receptors and the circumstances of their expression in health and in airways diseases in human airway mucosa require further exploration.

## 5. Apoptosis in Normal Airway Epithelium

Apoptosis has an important, beneficial regulatory role in the normal airway epithelium. As noted previously, it provides a mechanism to remove damaged cells without provoking an inflammatory response. An additional beneficial role is, along with cell proliferation, regulating the number of epithelial cells. The cell cycle rate in resting mammalian large airway epithelium *in situ*, expressed as the proportion of dividing cells counted in thymidine incorporation assays, is <1% [73, 74]. Corresponding rates of apoptosis, as judged by labeling for an apoptosis marker (e.g., TUNEL), are also low in normal mouse [75] and human [76, 77] airway mucosa. Exogenous and endogenous signals that stimulate proliferation may also stimulate apoptotic pathways so as to counter survival signals and thus maintain epithelial cell homeostasis [78].

One potential problem in measuring rates of apoptosis in airway epithelium, either normal or in disease states, is that dying epithelial cells may slough into the airway lumen prior to the demonstration of classic apoptosis markers by morphological methods. Detachment followed by cell death, termed *anoikis* (for a review see [79]), may lead to underestimation of rates of apoptosis in airway epithelium, though other markers of damage, such as focal gaps, denudation, or expression of markers such as the epithelial growth factor receptor or c-erbB-2 [80, 81], are still present. Dead (apoptotic or necrotic) epithelial cells in sputum degrade rapidly and may not be recognized or counted. One recent study collected sputum from marathon runners and then examined the number of apoptotic epithelial cells by TUNEL labeling followed by histologic examination. Their study demonstrated an ability to detect a significant number of positive cells [82]. This study suggests technical feasibility to examine the sputum. Another study examined the expression of *DAP kinase*, noted to have a role in TNF-*α*, Fas, and interferon-gamma- (IFN-*γ*-) induced apoptosis [83, 84] and in oncogenic transformation of cells [85], and in epithelial cells present in the sputum of a cohort of cancer patients, and correlated this to changes in expression in airway epithelium collected by bronchial brushing [86]. This study suggests that surrogate markers might be used to examine the presence of apoptotic epithelial cells and to examine activation or expression of pathways that might initiate cell death.

Another difficulty of detecting apoptotic cells in either normal or diseased tissue underscores the purpose of apoptosis: that of rapid, efficient execution, and clearance of damaged cells (now apoptotic bodies) so as to prevent aggravation of inflammation [87, 88]. Removal of apoptotic epithelial cells in the airway (whether by shedding into the lumen or by phagocytosis) may be accompanied soon after by proliferation and/or phenotype shifting of neighboring cells to replace the lost cells. Indeed, both apoptosis and proliferation may be increased in diseases such as asthma [53, 89]. Demonstration of significant numbers of apoptotic cells might not only suggest significant injury and response to injury, but may also suggest a defect in clearance of apoptotic cells (reviewed in [90]), a finding that, for example, may be of importance in emphysema [87]. Accumulation of apoptotic bodies may exert direct inhibitory or damaging effects on neighboring cells and may lead to continued inflammation [15], though this has yet to be specifically demonstrated in airway mucosa.

Therefore, particularly in disease states, the true proportion of apoptotic cells may be underestimated by morphological methods that depend on the continued presence of the dead or dying cell.

Since airway epithelial cells have the CD95 receptor, it follows that ligating that receptor should activate pathways that lead to cell death. Both in primary cells and in cell lines, this is clearly true [20, 50]. However, of interest is not that epithelial cells die in response to CD95 ligation, but that in most culture systems using primary cells collected from normal subjects or in cell lines, maximal activation of this receptor elicits death in only 10–20% of the cells present over a 24 to 48 hr time period. This is in sharp contrast to studies done in lymphocytes, eosinophils, and other inflammatory cells, in which CD95 ligation generally elicits >80% cell death in less than 8 hr (as several of many examples, see [64, 91, 92]). One might ask, if epithelial cells express both ligand and receptor, why one epithelial cell does not kill its neighbor? The answer may be that epithelial cells, at least under normal conditions in the airway (or in standard culture), are relatively resistant to CD95-induced death signaling, either by an inability to cluster and activate the receptor trimer required for FADD activation or further along the signaling pathway. This further suggests two possibilities. The first is that either a second “costimulatory” signal is required for maximal killing. No such signal has been demonstrated in airway epithelium, but signals that sensitize cells to death receptor stimulation are seen in other systems. For example, IFN-*γ* and TNF-*α* sensitize human endometrial stromal cells, normally apoptosis resistant, to CD95-mediated cell death due to an upregulation of CD95 expression [93], and interleukin (IL)-10 protects mouse intestinal epithelial cells from Fas-induced apoptosis by downregulating Fas expression and regulating the expression of death domain components [94].

A second possibility is that a change in the environment elicits a reconfiguration of the receptor that makes it more amenable to activation. Data to support such a hypothesis in airways are lacking, but, in other systems, it is clear that CD95 receptor regulation in turn regulates the ability to switch on apoptosis. For example, expressing a mutant CD95 that lacks a death domain, and therefore cannot initiate DISC formation, blocks apoptosis ordinarily elicited with an anti-CD95 antibody, even as the receptor trimers aggregate [95]. Expression of the cellular FLICE-like inhibitory protein (c-FLIP), a component of the death-effector domain, can either inhibit or activate both CD95 and other death receptors dependent on context (reviewed in [24]), but this regulator has yet to be described in airway epithelium.

In the normal airway epithelium then it is clear that apoptosis, like proliferation, is tightly regulated to the point that it is uncommonly seen as best as can be detected with current methods. Epithelial cells can respond to death stimuli in the culture environment but even then there is relative resistance. Understanding the “default” setting of “no death” may help us to understand compensatory responses and disease states in which apoptosis clearly is activated.

## 6. Apoptosis as a Mechanism to Maintain Epithelial Cell Homeostasis

Both in disease states such as asthma and COPD and after various environmental exposures, significant goblet cell hyperplasia (GCH) may be seen [96–98] in airway mucosa. Cytokines such as IL-13 and IL-4 can stimulate phenotype shifting of large airway basal [99] or small airway epithelial (Clara) cells [100] to mucoid cells by inducing mucin expression [99–104]; two separate reports suggest that ciliated cells may also shift to a mucoid phenotype under certain conditions [105, 106]. The bacterial wall inflammatory factor lipopolysaccharide (LPS) also can induce GCH in cell culture models of epithelial cell phenotype shifting [107, 108] as can irritants such as cigarette smoke [109]. There may also be an increase in the total number of epithelial cells over time with most of the new cells manifesting a mucoid phenotype [110]. Resolution of goblet cell hyperplasia is associated with downregulation of mucin protein expression and either phenotype shifting of these cells to a Clara or serous cell phenotype [100] or absolute reduction of goblet cell numbers by apoptosis [111–113].

The appearance of new goblet cells in GCH may be due to an inhibition of apoptosis. Harris and colleagues [110] demonstrated increased expression of Bcl-2 in mucous cells after LPS instillation in Norway rats; Bcl-2 positive mucous cells decreased to normal levels just prior to resolution of GCH. In their study, stable overexpression of Bcl-2 increased LPS-induced GCH compared to wild-type mice [110]. A similar expression of Bcl-2 and GCH was seen in nasal epithelium after ozone exposure in rats [114]. Resolution of GCH may require more than downregulation of Bcl-2: expression of the proapoptotic mitochondrial regulator Bax, which can heterodimerize with Bcl-2 and block its function [115], is associated with the loss of goblet cells in the resolution phase of GCH, and treatment with agents such as IFN-*γ*, which increases Bax expression, is associated with increased clearance of goblet cells [116], even as treatment with an anti-Fas antibody fails to clear these cells [113]. Other modulatory pathways such as that coupled to the epidermal growth factor receptor (EGFR) also may modulate GCH induced by either allergen exposure in cultured H292 epithelial cells [117] or Sendai virus exposure in mice [118], such that blocking EGFR signaling induced apoptosis in goblet cells.

These studies serve as one striking example of how apoptosis may be intimately involved in regulating the phenotype and presence of airway epithelial cells in response to environmental perturbations. Both the appearance and disappearance of goblet cells may require (resp.) inhibition or initiation of apoptosis. Both offer potential therapeutic checkpoints to drive the airway mucosa towards a more homeostatic model in response to chronic illness.

## 7. Apoptosis in Airway Epithelium in Disease

Epithelial cell apoptosis is a feature found in damaged and inflamed airways. While increased apoptosis is thought to contribute to airway damage and pathogenesis, it may also be a consequence of reparative processes that attempt to remove dead, dying, or damaged cells.

Discussion of epithelial cell apoptosis in airways diseases is hampered by methodological problems. In addition to the issues of detachment of apoptotic cells into the airway lumen and the clearance of apoptotic bodies by professional phagocytic cells, it can be difficult to distinguish apoptotic from necrotic cells [14] in airway and lung biopsies. Further, a measurement of apoptosis at a single time point (e.g., via endobronchial biopsy) may not reflect either the state of damage in that airway at that time point, of the disease state elsewhere in the lung at that time point or of the course of the disease over time. Repeated measurements of epithelial cell apoptosis *in vivo* over time are currently not possible, as there are no specific markers to be found in sputum (noting the recent study of Chimenti et al. [82] that suggests at least the possibility of identifying TUNEL-positive epithelial cells in sputum collected from asthmatic subjects), bronchoalveolar lavage fluid, or exhaled breath condensate, and repeated biopsy of the airways, in addition to methodological problems, has obvious limitations in terms of patient access and risk. The discussion that follows for each disease then is based on limited *in vivo* studies supplemented by mouse and culture models.

### 7.1. Asthma and Airway Inflammation

Asthma has been recognized since antiquity. The word derives from the Greek meaning “short of breath.” By the end of the 19th century, Henry Hyde Salter had recognized the appearance of an asthmatic airway with inflammation and hypertrophy of smooth muscle [119], and Sir William Osler had described the relation of allergy, hay fever, familial predisposition, childhood onset, the presence of Leyden crystals and Curschmann spirals in sputum, and paroxysms to asthma [120]. In the early to mid 20th century, asthma was seen as a disease of intermittent bronchospasm and treated with various bronchodilators. But from the 1920s, inflammation was recognized as pathogenetic to asthma (for a review see [121]), and, more recently, the concept of “remodeling”, a description of a chronically inflamed, narrowed asthmatic airway with smooth muscle hypertrophy, a thickened basement membrane, and—importantly for this discussion—chronic, persistent, focal to widespread epithelial damage along with goblet cell hyperplasia [98]—as an end result of inflammation, has defined the disease process [122, 123].

The epithelium is a target of inflammatory and physical insults in both acute asthmatic inflammation and in chronic asthmatic remodeling [1]. Epithelial injury is common even when the clinical state of asthma is mild [123, 124] and is persistent over time [1]. Epithelial damage correlates with airway hyperreactivity and may be seen in newly diagnosed asthma [125, 126]. Epithelial damage is more than a manifestation of injury: it is an effector of the airway inflammation that marks chronic asthma [127–129]. Epithelial injury leads to disordered regulation of submucosal myofibroblasts and fibrinogenesis [130, 131] and release of chemokines such as IL-8 and eotaxin [132, 133]. Mucosal damage may be extensive in severe or fatal asthma [134] but is more focal in mild-moderate asthma [125, 135].

#### 7.1.1. Human Studies

**Figure 3:**
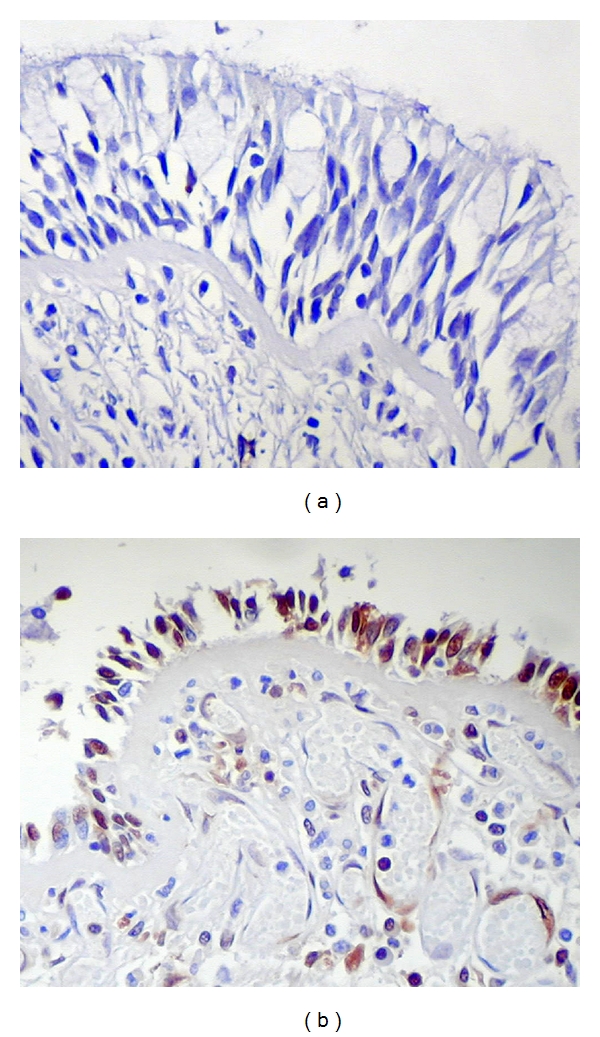
Demonstration of apoptotic airway epithelial cells, as demonstrated by TUNEL stain for single-strand DNA nicking using a peroxidase method (brown, with hematoxylin counter label), in an endobronchial biopsy of a normal subject (a) and of a subject with chronic, persistent asthma (b). Note the substantial difference in the height of the epithelium and the gaps in the epithelium at the basement membrane between the two biopsies, and the loss of ciliated cells in asthmatic biopsy. The appropriate controls (not shown here) do not label. Original magnification, 200x. From the author's laboratory.

Epithelial cell apoptosis is difficult to assess in human asthma, both for morphological issues such as sampling bias and the aforementioned risk of underestimating apoptosis in damaged airways, and issues due to the variable nature of the disease and variable methods used to classify patients. Studies of apoptotic markers in endobronchial biopsies collected from asthmatic subjects have shown variable results: for example, Vignola et al. [77] examined TUNEL, Bcl-2, and p53 labeled in a cohort of asthmatics, either untreated or treated with inhaled or oral corticosteroid (CS) agents, and in control subjects. In this study no control subject, and few asthmatic subjects, had any TUNEL-positive cells in the epithelial layer; while p53 was not expressed in any group, subjects with asthma, treated or not, had a higher number of cells labeling for Bcl-2 versus control. In contrast, a study by Cohen et al. [89] examined both apoptosis and proliferation in bronchial biopsies collected from normal subjects and subjects with mild or severe asthma. In this study, there was a greater number of TUNEL-positive cells in biopsies of severe asthmatics compared to controls, along with decreased Bcl-2 expression and increased proliferation as noted by labeling for the marker Ki-67. These two studies may be reconciled on the basis of disease severity, as the Cohen study did not demonstrate an increase in apoptosis in mild asthmatics [89]. Yet another study by Trautmann et al. [53] demonstrated apoptotic epithelial cells in a small cohort of patients with mild, persistent asthma, as demonstrated by TUNEL and Hoechst labeling on endobronchial biopsies. In our own laboratory, apoptotic epithelial cells can be demonstrated clearly in endobronchial biopsies of subjects with chronic, persistent asthma; such labeling is almost never seen in biopsies collected from normal volunteer subjects (Figure 3).

Therefore, as best can be demonstrated to date, there are relatively few apoptotic cells present in the airway epithelium of mild asthmatics; more significant apoptosis and proliferation are seen in asthma and may be related to both chronicity and severity.

#### 7.1.2. Effect of Corticosteroids

Corticosteroid therapy in asthma does not necessarily reverse epithelial damage seen in asthma. Corticosteroid treatment clearly suppresses inflammation in a large majority of asthmatic patients [136–139] and in mouse asthma models of allergen-induced airway inflammation [137, 140], though it is clear that corticosteroids do not alter the natural history of asthma [139]. In addition to the effects on the number and function of inflammatory cells that ordinarily infiltrate the airway mucosa in chronic asthma, CS treatment also inhibits the release of inflammatory mediators secreted by epithelial cells such as RANTES [141], GM-CSF [142], and eotaxin [143].

However, the role of corticosteroids on epithelial cell survival and death is less clear. That some asthmatic subjects have improved airway mucosal integrity and epithelial cell anatomy following treatment with inhaled CS is unequivocal: for some subjects, clinical improvement is accompanied by evidence of restoration of normal epithelial anatomy as demonstrated on endobronchial biopsies [144]. Further, CS treatment of cultured epithelial cells may inhibit cell death induced by cytokines such as IFN-*γ* or TGF-*β*. Dexamethasone at concentrations of 1 mM inhibits IFN-*γ* induced cell death in the A549 peripheral lung adenocarcinoma cell line, perhaps by inducing expression of hIAP [145]. Contradictory reports suggest that TGF-*β* can induce epithelial cell apoptosis that is blocked by concurrent treatment with budesonide [146] or can prevent epithelial cell apoptosis that is induced by dexamethasone [147].

Balancing these studies are reports that CS treatment of cultured human airway epithelial cells and cell lines may induce apoptosis [50, 147–149]. Either dexamethasone or budesonide in concentrations of 1–10 *μ*M in culture, a dose that when adjusted for airway surface area and sol volume calculates to the high end of the point concentration of an inhaled corticosteroid on epithelium *in vivo*, elicits caspase-mediated apoptosis that requires disruption of mitochondrial polarity and release of cytochrome c [50]. Overexpressing either Bcl-2 or Bcl-xL inhibits CS-induced apoptosis in this model. A follow-up study demonstrated that dexamethasone treatment in Balb-c mice for 3 days to 4 weeks also elicited increased epithelial cell apoptosis, as measured by TUNEL labeling and labeling for the 85 kD fragment of polyadenine ribopolymerase (PARP), a nuclear enzyme cleaved early in apoptosis [75]. One additional study also suggests that concurrent treatment of cultured human airway epithelial cells and cell lines with the beta-adrenergic agonists albuterol or formoterol can block CS-induced apoptosis [149]—this may provide one potential explanation as to why relatively few apoptotic epithelial cells are seen in the airways of asthmatic subjects, as most of these subjects receive such agents as part of their antiasthma controller therapy.

#### 7.1.3. Allergen Exposure, Asthma, and Epithelial Cell Apoptosis

Mouse models of airway inflammation induced by exposure to allergen are a time-honored, useful model to explore the effects of an allergen on airway structure and function and, unlike in human studies, permit careful assessment of mechanism and function. The classic model involves the sensitization and challenge to ovalbumin (OVA) [150], followed by collection of tissue, BAL fluid, or other samples at key time points following challenge. Genetic manipulation of the mouse, adoptive transfer of selected lymphocytes, or other treatments can modify airway inflammation. From these many studies, a clear picture of murine airway inflammation has developed that has informed our understanding of human airway inflammation in asthma.

Two laboratories have demonstrated increased epithelial cell apoptosis after OVA challenge in mice. Truong-Tran et al. [151] examined apoptosis markers such as caspase-3 activity in an OVA model in Balb/c mice. Allergen challenge elicited airway inflammation and airway hyperresponsiveness, as expected, and also an increased number of apoptotic bodies in the airway mucosa and increased immunolabeling for activated caspase-3; both were increased by concurrent dietary depletion of zinc, a factor that the authors suggest may be protective against epithelial damage.

A second study from our laboratory [75] examined epithelial cell apoptosis after OVA challenge with and without concurrent corticosteroid therapy. This study grew from an earlier observation (discussed above) that corticosteroid treatment could induce apoptosis in cultured primary human airway epithelial cells and cell lines [50]. We hypothesized that corticosteroids could cause cell death of airway epithelium *in vivo*, and the resulting loss of epithelial cells might explain in part the damage to and denudation of the airway mucosa in chronic asthma, despite control of other markers of inflammation. To test this, Balb/c mice were sensitized and challenged to OVA, and both sensitized mice and control, unsensitized mice were treated at selected time points with dexamethasone in doses calculated to be at the high end of a therapeutic regimen for asthma. Allergen challenge also elicited increased TUNEL and p85-PARP labeling over the first 14 days after challenge, and this was not decreased by concurrent corticosteroid treatment.

These two papers have made clear the association of epithelial cell apoptosis and allergen-induced airway inflammation. Human studies to examine the association and to perhaps demonstrate causality of allergen challenge on epithelial cell apoptosis, however, have not been done to date. One paper by Robertson et al. [69] examined the expression of the TNF family mediator TRAIL, which can induce apoptosis and its receptors DcR2, DR4, and DR5 following segmental allergen challenge by bronchoscopy in a cohort of asthmatic and nonasthmatic subjects with ragweed allergy. In asthmatic subjects, instillation of ragweed into the airways elicited increased epithelial TRAIL labeling within 2 days along with decreased immunostaining of DR4 and DR5 in eosinophils and macrophages. However, no labeling of endobronchial biopsies to examine epithelial cell apoptosis was done.

#### 7.1.4. Airway Epithelial Cell Damage and Apoptosis in Response to Exercise and to Cold/Dry Air

A substantial proportion of asthmatic patients demonstrate worsening symptoms and airway inflammation as a result of exposure to cold, dehumidified air associated with exercise [152–154]. Cold air challenge and hyperpnea both are clinical and laboratory models used to induce bronchoconstriction in susceptible subjects with airway hyperreactivity [155, 156]. Athletes, even those not suspected of having asthma, may also have airway inflammation in response to cold air exposure [157–159]. Even in more moderate environments such as swimming or rowing in which there is substantial hyperpnea, elite athletes may have inflammatory cells present in induced sputum and in airway lavage [159, 160]. Airway epithelial damage in athletes may be seen: for example, in a cohort of nonasthmatic long-distance runners, increased numbers of shed bronchial epithelial cells and of TUNEL-positive apoptotic epithelial cells were seen in induced sputum after a half-marathon race [82]. These findings have been explored in a mechanistic model in mice, in which lung sections collected from sedentary and endurance-trained mice were examined [161]. Compared to sedentary mice, the bronchiolar epithelium of endurance-trained mice demonstrated a progressive loss of ciliated cells and both increased apoptosis and increased proliferation, along with infiltration of CD45+ leukocytes into the mucosa. These changes may represent an adaptive response to increased ventilation during exercise.

In summary, asthma, asthma treatment, and Th2-mediated airway inflammation are associated with central airway epithelial cell apoptosis. The significance of this cell death is not yet clear, nor is it clear whether epithelial cell apoptosis represents a compensatory, adaptive response to clear damaged or dying cells or represents a maladaptive, pathologic response that over time contributes to the epithelial injury and airways remodeling process that characterizes chronic asthma.

### 7.2. Viral and Bacterial Infection

Viral infection of the airways is increasingly demonstrated as a leading cause of exacerbations of clinical asthma, particularly in childhood [162]. Infections with viruses such as adenovirus (AdV), rhinovirus (RV), respiratory syncytial virus (RSV), influenza virus, and parainfluenza virus (PIV) occur early in the lower respiratory tract, are association with exacerbations and hospitalizations for asthma [163, 164], and are recognized as an important risk factor in early childhood for the development of persistent asthma later in life [165, 166]. Patients with asthma with frequent viral-induced exacerbations have worse pulmonary function and a worse clinical course than patients without such frequent infection [167], a finding that may first develop in infancy [168]. Frequent viral infection in infancy may alter Th2 responses [169], particularly when combined with allergen exposure [170]. The airway epithelium is a central target of viral infection and replication, and epithelial cell damage, release of cytokines, and other factors. Apoptosis then can be seen as a central part of host defense; viruses may use apoptosis to subvert host defense for their own survival. One broad method for doing so is for a virus to provoke apoptosis of the infected cell. The potential advantage of this is that it may facilitate viral egress after replication and thus spreading and survival of the virus (reviewed in [171]).

Infection with RSV can induce apoptosis in epithelial cells. This was clearly seen in the study of Kotelkin and colleagues [172] in which RSV infection of cultured primary tracheal or small airway epithelial cells, or cell lines, elicited apoptosis that was associated with both caspase-8 (receptor mediated type I) and caspase-9 (mitochondrial associated type II) activation and with expression of both pro- and antiapoptotic Bcl-2 family members. Interestingly, RSV also induced TRAIL expression and expression of both the DR4 and DR5 receptors, not seen in normal, uninfected cells. A more recent study demonstrated that RSV infection was associated with recovery of soluble TRAIL in airway lavage fluid and the expression of the TRAIL receptors R1 and R2 in the airway epithelium of children with RSV infection and mechanical ventilation [72]. These findings suggest that TRAIL may contribute to epithelial injury during severe RSV infection. A third study examined the apoptosis-inducing effect of RSV on primary nasal, tracheal, and bronchial epithelial cells grown in culture. In this study, RSV elicited apoptosis that was increased after knockdown of endogenous nerve growth factor [173].

Adenoviral infection also can induce apoptosis in epithelial cells. In a guinea pig model of AdV infection, Singhera and colleagues [71] demonstrated increased, time-dependent apoptosis, as measured by detection of the p85 fragment of PARP, along with expression of the DR4 and DR5 receptors. Interestingly, corticosteroid treatment delayed apoptosis in cultured epithelial cells following AdV infection and allowed increased viral particle production. In a chronic infection model, infection plus sensitization and challenge with ovalbumin led to increased numbers of apoptotic epithelial cells; again, corticosteroid treatment decreased the number of apoptotic cells. These experiments suggest that corticosteroid use may actually contribute to decreased viral clearance and thus prolonged infection.

Another virus potentially important to asthma is RV. While RV infection is less toxic to epithelial cells in different models, it can induce apoptosis via activation of caspase-9 in cultured, nonpolarized epithelial cells in culture [174]. However, apoptosis was not seen in RV-infected, polarized, differentiated epithelial cells, even as RV infection disrupted the epithelial barrier function as measured by transepithelial resistance [175].

Influenza A infection elicits an inflammatory airway response with epithelial cell secretion of cytokines and chemokines. One study has examined influenza-A-infection-induced apoptosis in the H292 human mucoepidermoid bronchiolar carcinoma cell line. In this study, Brydon et al. [176] demonstrated that inhibiting apoptosis using caspase inhibitors increased chemokine secretion, suggesting that the observed inflammatory response in influenza would be greater if cell death did not occur. Another study demonstrated that, in H292 cells, influenza infection elicited apoptosis by upregulating mitogen-activated protein kinase signaling pathways that include c-Jun and p38-MAPK [177]. The avian H9N2 influenza virus elicits apoptosis in primary, polarized, differentiated airway epithelial cells with activation of caspase-9 and release of mitochondrial cytochrome c [178]. In this study, IFN-*β* was antiviral and antiapoptotic, which fits the known role of interferons in blocking viral infection [171]. In this light, a study by Chan et al. demonstrated that more differentiated airway epithelial cells in culture were more resistant to H5N1 influenza infection [179]; the authors noted that an undifferentiated epithelium, as might be seen following damage due to virus or other agents, could be easily infected. Examining apoptosis in both undifferentiated or growing epithelial cells versus a tightly organized differentiated epithelium with a highly polarized and tight barrier may be required to understand how influenza and other viruses use cell death to their advantage in clinical infection.

Viral infection also may suppress apoptosis so as to ensure their replication and survival by preventing the death of the cell. While cytomegalovirus and certain herpesviruses are known to inhibit apoptosis (reviewed in [171]), no data suggest that such suppression occurs after viral infection of airway epithelium.

Taken together, these studies suggest that infection with viruses that commonly cause respiratory disease such as RSV, AdV, and influenza elicit apoptosis of airway epithelium. The interplay between epithelial damage and loss, inflammation, and viral replication is not yet clear from these studies. Studies are needed to demonstrate whether blocking apoptosis in viral infection leads to clear changes in the inflammatory state and epithelial damage, and better studies in human subjects, particularly children, are needed to demonstrate the association of epithelial cell damage, loss, and apoptosis with ongoing airway inflammation and loss of asthma control. Though challenging, such studies will provide important answers as to whether inhibiting apoptosis in the setting of viral infection leads to better, or worse, clinical outcomes.

### 7.3. Cystic Fibrosis

Cystic fibrosis (CF) is a multisystem, genetic disease caused by mutations in the gene encoding CFTR, which leads to decreased chloride secretion and increased sodium absorption, with resulting decreases in lumenal liquid, in airways, the pancreas, and other organs lined by epithelial cells (reviewed in [180]). In airways, this leads to dehydration of airway mucous with resulting poor mucous clearance, infection, particularly with organisms such as *Pseudomonas aeruginosa* and *Staphylococcus aureus*, inflammation, and, over time, bronchiectasis and fibrosis of the airway [180, 181].

Clearly the epithelium is central to disease pathogenesis in CF, and airway inflammation along with damage to and loss of epithelial cells are prominent in established bronchiectasis and airway fibrosis in this disease [182]. Over the past decade, apoptosis as one of several mechanisms for airway epithelial cell loss in CF airways has become recognized.

Expression of CD95 and CD95L, along with markers of apoptosis such as DNA fragmentation, was first reported by Durieu et al. [52] in a study of surgical lung lobectomies obtained from a small cohort of CF patients. In this study, CD95L expression was markedly increased in bronchial epithelial cells, and TUNEL labeling demonstrated apoptotic cells both in the mucosa and in submucosal gland epithelial cells. Harris et al. [110] demonstrated increased Bcl-2 presence in airway epithelium in lung blocks collected from patients with CF compared to normal airways; this was most prominent in goblet epithelial cells. Bcl-2 expression in this setting may be a general, compensatory response to permit survival or may permit preferential development of goblet cells that then would secrete increased mucous into the CF airway.

Apoptosis of CF-derived epithelial cells in culture also has been demonstrated. Ligating CD95 induces apoptosis (as expected) in normal tracheal epithelial cell lines but increased apoptosis in the CF-derived cell line CFT-2 [183]. The *Pseudomonas aeruginosa* strain PAO1 induces apoptosis, as demonstrated by TUNEL labeling, in about 10% of primary epithelial cells over 8 hr of exposure but 50% in the 9HTEo-transformed cell line that lacks tight junctions [184]; treating another transformed cell line, 16HBE14o- that has high-quality tight junctions, with EGTA made them more susceptible to PAO1-induced apoptosis. In contrast, stable transfection of a constitutively active variant of the regulatory R-domain of CFTR did not alter rates of apoptosis elicited by PAO1, nor did PAO1 elicit significant apoptosis in *cftr* ^−/−^   mice [184]. These data suggest that the intrinsic loss of CFTR is less important in epithelial cell survival than morphologic changes such as barrier integrity. This conclusion is challenged by a more recent study examining apoptosis after airborne particulate matter (PM) exposure in two epithelial cell lines, IB3-1 that contains compound heterozygote delta F508 and W1282X nonsense CFTR mutations, and S9 that is derived from the IB3-1 cell line with the CF phenotype corrected by transfection with wild-type adenoassociated viral CFTR. Upon exposure to PM in a standard model for 1 hr, IB3-1 cells had a rate of apoptosis greater than six fold higher than that seen in S9 cells; this was associated with disruption of mitochondrial polarity and activation of caspase-9 and blocked by overexpression of Bcl-xL [185]. Increased susceptibility to oxidative stress may account for the effect of PM in this model, and oxidative-stress, a known inducer of apoptosis in other systems, may also have other effects that induce cell death in CF airway epithelium. Ahmad and colleagues [186] examined the expression and activity of sarcoendoplasmic reticulum calcium ATPase (SERCA), a regulator of calcium homeostasis, in normal and CF epithelium and tissue. SERCA2 expression was decreased both in airway biopsies and in differentiated, polarized cultured epithelial cells from CF subjects and in CF epithelial cell lines, and expression of the Δ508-mutated CFTR in cell lines led to decreased SERCA2 expression. SERCA2 displaces Bcl-2 from the endoplasmic reticulum [187], and silencing of the SERCA2 gene enhanced epithelial cell death due to oxidative stress and to treatment with TNF-*α* [186].

*Pseudomonas* infection may also provoke epithelial cell apoptosis by inducing the expression of cationic innate host defense peptides that injure the host. One such cationic peptide is LL-37, the predominant cleavage product of human cationic antimicrobial peptide (hCAP)-18, the human cathelicidin (reviewed in [188]). LL-37 is secreted by both neutrophils and epithelial cells and is upregulated in response to infection and inflammation [188, 189]. Expressing LL-37 in murine lung enhances the clearance of pulmonary *Pseudomonas aeruginosa *[190]. LL-37 can induce apoptosis in both primary cells and in cell lines [191–193] and does so via activation of the intrinsic pathway with release of cytochrome c and activation of caspase-9 [193]. Interestingly, in the latter study, cell death required the presence of both the cationic protein and live bacteria, and either alone was incapable of inducing apoptosis [193]. The effect of other cationic proteins such as the eosinophilic cations or that of synthetic cationic proteins such as polylysine have not been examined in the context of airway epithelial cell death.

Taken together, these studies show that epithelial cell apoptosis may be present in CF airways and that CF epithelial cells are perhaps more susceptible to apoptotic cell death following oxidant stress, bacterial infection, and perhaps to host defense factors (LL-37 as one demonstrated example). The relative contribution of apoptosis to epithelial damage in CF airways, as well as the contribution overall to CF pathophysiology, is yet to be defined.

### 7.4. COPD

Chronic obstructive pulmonary disease (COPD) arises as a result of the inhalation of noxious stimuli to the lungs, most commonly cigarette smoke. Damage to the central airways (chronic bronchitis) and to the peripheral lung (emphysema) commonly occurs. Several mechanisms contribute to the pathogenesis of COPD, including influx of inflammatory cells into the lung, disruption of the balance between proteolytic and antiproteolytic activity, and oxidative stress [194].

Some recent studies have suggested increased levels of apoptosis in peripheral lung endothelial cells [195, 196] and alveolar inflammatory cells [197, 198] (also reviewed in [199]). It is surprising, given the levels of oxidative stress and the toxins inhaled in cigarette smoke, that apoptosis has not been investigated rigorously in central airway epithelial cells. One recent study from Korfei et al. [200] examined markers of apoptosis in explant tissue obtained from a small cohort of patients with COPD, idiopathic pulmonary fibrosis, or donated lung and demonstrated no evidence of such markers in COPD. Another study examined the presence of apoptosis in central airway epithelial cells collected by endobronchial brushing of 2–4 mm airways from 20 normal subjects and 23 subjects with COPD [201]. Several assays to assess apoptosis were done to minimize the risk of technical artifact skewing the results. Small airway epithelial cells collected by brushing from the patients with COPD had increased labeling for the presence of apoptosis by each assay; this was not affected by either the age of the subject or by smoking status. A recent study in which mice were exposed either acutely or chronically to ozone demonstrated that chronic exposure led to both central and peripheral airway epithelial cell apoptosis as measured by immunoreactivity to caspase-3 and the apoptosis protease activating factor-1 (APA-1) [202]. How apoptosis in the more central airways relates to the genesis of chronic bronchitis or airway inflammation is not yet known.

Peripheral alveolar epithelial cells in smoking subjects with COPD also show evidence of apoptosis markers such as p53 [203] or TUNEL [198, 204]. It is interesting to speculate that such cell death may contribute to the pathogenesis of emphysema, but no firm data has emerged.

### 7.5. Interstitial Lung Disease

Interstitial lung diseases comprise a spectrum of illnesses that have at their core fibrotic damage to the peripheral lung. One of these, idiopathic pulmonary fibrosis (IPF), is a progressive disease in humans that has, as central to its pathogenesis, injury to small airways and alveolar epithelial cells [205, 206]. Over time continued injury and reaction to injury lead to impaired epithelial repair, phenotype shifting of fibroblasts to myofibroblasts, matrix deposition, and scarring. Several types of injuring agents such as oxidative stress have been postulated as causes of the initial injury [207], even though antioxidant therapy has but modest benefit in these patients [208, 209].

Apoptosis is clearly recognized in the alveolar epithelium in both human tissue samples of patients with IPF [210, 211] and in mouse models of lung fibrosis [212, 213]. Increased expression of Fas [214–216] and of apoptosis signaling pathway intermediates such as p53 [210, 216, 217] are seen which may help explain the loss of epithelial cells. In contrast, damage to and apoptosis of more central airway epithelial cells are not seen [211].

Central airway epithelial cell apoptosis is not a prominent feature of other interstitial lung diseases, such as sarcoidosis or the collagen-vascular-associated lung diseases, as reported to date.

### 7.6. Lung Transplantation

Lung transplantation (LT) remains the best hope for selected patients with end-stage lung diseases. Chronic allograft rejection, clinically manifested as bronchiolitis obliterans syndrome (BOS) and pathologically as obliterative bronchiolitis (OB), remains a major limitation to long-term survival: BOS occurs in 40–60% of lung transplant recipients within 4 years [218, 219] and is the leading cause of death after the first year [220, 221]. Allograft rejection is mediated mainly by recipient alloreactive CD4+ and CD8+ effector T lymphocytes [222, 223] developed against mismatches in HLA class I and II antigens of the epithelial cells in the allograft [224–226]; these in turn may be suppressed by regulatory T suppressor lymphocytes that promote tolerance [227, 228].

There are few reports of epithelial cell apoptosis in the transplanted human lung. An early study suggested that in transplanted lungs in which obliterative bronchiolitis was present, there were increased numbers of TUNEL-positive cells in large airway, small airway, and alveolar epithelial cells, whereas such labeling was virtually absent in transplant lung specimens that had no evidence for OB [229]. An elegant study of ischemia reperfusion in the transplanted lung collected samples from peripheral donor lungs during cold ischemia, warm ischemia, or after graft reperfusion at the time of implantation. In this study, TUNEL labeling demonstrated significant alveolar but not more central epithelial cell apoptosis during the time of reperfusion but not during either ischemia periods [230].

Animal lung transplantation models provide important data concerning the role of apoptosis in obliterative bronchiolitis and in ischemia-reperfusion injury. Increased numbers of apoptotic epithelial cells are seen prior to sloughing and obliterative bronchiolitis in the mouse heterotopic tracheal transplant model [231] and in a similar model in swine [232], though the degree of apoptosis is modest compared to the subsequent loss of epithelial cells, suggesting that other processes such as necrosis or autophagy are as or more important. A more recent study examines epithelial cell apoptosis in an orthotopic mouse tracheal transplant model with preservation of ventilation through the transplanted airway [233]. In this study, ventilation was permitted or occluded through the transplanted trachea, and morphometric and immunohistochemical measurements were made 28 days after transplant. Epithelial cell morphology was better preserved in the ventilated group with more numerous ciliated cells, and TUNEL-labeling was substantially less in these allografts, ~12%, versus those that were not ventilated, ~66%. Evidence for obliterative airways disease was present in the nonventilated allografts suggesting an association between OB and epithelial cell apoptosis. Other studies suggest that airway obliteration characteristic of the heterotopic tracheal allograft model does not occur in orthotopic allografts and that recipient epithelial cells migrating into the donor graft may actually prevent obliteration [234–236]. The role of apoptosis in this process is not known.

One intriguing study examined the role of treatment with an endothelin-A/B receptor antagonist, SB209670, to block apoptosis following ischemia-reperfusion injury in a dog model of lung allotransplantation [237]. Blocking the endothelin receptors led to lower numbers of apoptotic epithelial cells in the transplanted peripheral airways and alveoli as assessed by TUNEL labeling. This may be due to the ability of this antagonist to increase Bcl-2 expression [238].

One paper has examined epithelial cell apoptosis in a culture model using the KCC-266 cell line; treatment with an antibody that activates class I HLA responses (W6/32) elicits both proliferation and apoptosis over 48 to 72 hr [225]. However, the cell line is not characterized or described in this study or elsewhere, and it is not clear whether this is a central or alveolar-derived cell.

#### 7.6.1. Environmental Exposures

Environmental agents may disrupt the integrity and function of the airway epithelium. In addition to allergen exposure as noted above, various gasses and particles may elicit damage via initiation of apoptosis. One group of agents include the incompletely-combusted components of fossil fuels such as polycyclic aromatic hydrocarbons (PAHs). One such family of hydrocarbons, benzo[a]pyrenees, can elicit apoptosis in primary small airway epithelial cells, but not the peripheral A549 adenocarcinoma epithelial cell line, in culture [239], by increasing free cytosolic calcium levels [240].

Fine particulate matter also can induce apoptosis in CF-derived epithelial cells as previously noted [185]. Another study of the effect of fine particulate matter (PM2.5) on the L132 human lung epithelial cell line demonstrated that exposure-induced apoptosis by both receptor-mediated (caspase-8 activation) and mitochondrial (cytochrome c release and caspase-9 activation) pathways [241]. These fine particles are composed of a variety of inorganic and organic chemicals, including PAHs, and it is not clear which part of the particle is responsible. To make this more complicated, another study demonstrated that a water-soluble fraction of PM2.5 actually inhibits apoptosis induced in three different epithelial cell lines and in primary epithelial cells [242]; the authors suggest that blocking apoptosis may contribute to prolonged inflammation and impaired repair in airways after pollution exposure. In support of this, Rumelhard et al. [243] examined the expression and role of EGFR ligands such as amphiregulin, heparin-binding epidermal growth factor-like growth factor (HB-EGF), and TGF-*α* in primary airway epithelial cells and the 16HBE14o- cell line after exposure to PM2.5. Secretion of each ligand was seen, and each elicited expression of cytokines such as granulocyte-macrophage colony stimulating factor (GM-CSF). This study was subsequently replicated in primary airway epithelial cells using PM2.5 with different PAH and metal contents, demonstrating significant overlap between the groups suggesting that the exact composition of the particulate matter is less important [244]. Clearly, the effect of environmental agents on epithelial cell apoptosis, as well as the subsequent effect upon airway homeostasis, requires further investigation.

One interesting paper notes the effect of mechlorethamine, a functional analogue of vesicant sulfur mustard used in chemical warfare, on differentiated HBE1 cells in culture. In this model, mechlorethamine treatment elicited epithelial layer disruption with evidence of both cytotoxic and apoptotic changes in a concentration and time-dependent manner [245].

## 8. Conclusions

Apoptosis is an important regulator of epithelial cell survival in several important clinical diseases. In contrast to inflammatory cells which undergo apoptosis readily and quickly after an appropriate stimulus, epithelial cells have some innate resistance to similar provocation. Understanding this relative resistance to cell death may hold a vital clue for understanding the role of apoptosis in airway homeostasis and may also have important implications for diseases that are epithelial based but not touched on in this paper, such as lung cancer. While this paper has not considered the roles of necrosis and autophagy in epithelial cell death, the former especially may be as, or more, important in selected airway conditions. Nevertheless, apoptosis clearly is involved in epithelial cell death in several inflammatory airways diseases.

There is regional variation in the response to apoptotic stimuli: when directly assessed, small airways (bronchiolar) epithelial cells are more sensitive than those collected from larger bronchi or trachea. There is significant variation in the response to activation of death receptors and in receptor-mediated versus stress-activated (via mitochondrial polarity disruption) apoptosis. Again, the reasons for this are not understood.

Finally, apoptosis is required to return an inflamed airway, or one that is hyperplastic or metaplastic, to normalcy. The controlling mechanisms by which this occurs are still not understood. Recognizing when and how apoptosis is important to airway inflammation and how to turn that process to our advantage offers potential therapeutic targets for several airways diseases.
